# Reverse engineering gene regulatory network from microarray data using linear time-variant model

**DOI:** 10.1186/1471-2105-11-S1-S56

**Published:** 2010-01-18

**Authors:** Mitra Kabir, Nasimul Noman, Hitoshi Iba

**Affiliations:** 1 Department of Computer Science and Engineering, University of Dhaka, Dhaka, Bangladesh; 2 Iba Lab, Dept. of Electronics Engineering, Grad. School of Engineering, University of Tokyo, Tokyo, Japan

## Abstract

**Background:**

Gene regulatory network is an abstract mapping of gene regulations in living cells that can help to predict the system behavior of living organisms. Such prediction capability can potentially lead to the development of improved diagnostic tests and therapeutics. DNA microarrays, which measure the expression level of thousands of genes in parallel, constitute the numeric seed for the inference of gene regulatory networks. In this paper, we have proposed a new approach for inferring gene regulatory networks from time-series gene expression data using *linear time-variant *model. Here, *Self-Adaptive Differential Evolution*, a versatile and robust Evolutionary Algorithm, is used as the learning paradigm.

**Results:**

To assess the potency of the proposed work, a well known nonlinear synthetic network has been used. The reconstruction method has inferred this synthetic network topology and the associated regulatory parameters with high accuracy from both the noise-free and noisy time-series data. For validation purposes, the proposed approach is also applied to the simulated expression dataset of cAMP oscillations in *Dictyostelium discoideum *and has proved it's strength in finding the correct regulations. The strength of this work has also been verified by analyzing the real expression dataset of SOS DNA repair system in *Escherichia coli *and it has succeeded in finding more correct and reasonable regulations as compared to various existing works.

**Conclusion:**

By the proposed approach, the gene interaction networks have been inferred in an efficient manner from both the synthetic, simulated cAMP oscillation expression data and real expression data. The computational time of this approach is also considerably smaller, which makes it to be more suitable for larger network reconstruction. Thus the proposed approach can serve as an initiate for the future researches regarding the associated area.

## Background

*Gene Regulatory Networks *(GRNs) are the functioning circuitry in living organisms at the gene level. It is regarded as an abstract mapping of the more complicated biochemical network which includes other components such as proteins, metabolites, etc. The purpose of GRN is to represent the regulation rules underlying the gene expression. Understanding GRNs can provide new ideas for treating complex diseases and breakthroughs for designing new drugs.

Gene regulatory network reconstruction is currently a topic under heavy research in the computational biology field. The study of GRN is made much easier with the recent introduction of *microarray *technology. Using this method, expression levels of thousands of genes can be measured simultaneously, as they change over time and are affected by different stimuli. Thereby, it is possible to obtain a global view of the dynamic interaction among genes. But it is a great challenging problem to discover these networks of interacting genes that generate the fluctuations in the gene expression levels. Inference of GRNs based on microarray data is referred to as *reverse engineering *[1], as the microarray expression levels are the outcome of gene regulation. Mathematically, reverse engineering is a traditional inverse problem. The solution to the problem is, however, not trivial, as it is complicated by the enormously large scale of the unknowns in a rather small sample size. In addition, the inherent experimental defects, noisy readings, and many other factors play a role. These complexities call for heavy involvement of a powerful mathematical modeling together with reliable inference, which play an increasingly important role in this research.

Various types of models, namely, directed graphs, Boolean networks [2-4], Bayesian networks [5-7], various differential models describe gene regulation at various levels of detail and complexity and the model of choice is often determined by how much information the model tries to capture. The more information a model tries to infer, the more parameters are needed to learn, the more complex the model becomes. However, these potentially powerful formalisms are limited by their ability to handle noisy data, nonlinear gene regulations as well as their computational complexity. So, one has to hunt for such a computational approach that can overcome or at least alleviate the problems of the existing ones and thereby, be able to reflect the true regulatory networks. A particularly promising nonlinear model for this purpose is *S-system *proposed by [8] and [9] for the analysis of time series gene expression data. This model possesses a rich structure which is capable of capturing various dynamics of the complex regulation, and can be analyzed by several available methods. According to S-system model, traditional rate laws are approximated with a set of nonlinear differential equations in which the component processes are characterized by the following power-law functions:(1)

Here, n is the number of genes or system components and *X*_*i *_is the expression level of gene-i. The exponential parameters *g*_*i*, *j *_and *h*_*i*, *j *_are the interactive effect of *X*_*j *_to *X*_*i*_, which are also referred to as *kinetic orders*.

The gene network inference problem based on the S-system model is defined as an estimation problem of the S-system parameters (*α*, *β*, *g*, *h*). But the major disadvantage of the S-system is the large number of parameters to be estimated: 2*n*(*n *+ 1). Because the number of S-system parameters is proportional to the square of the number of network components, the algorithms must simultaneously estimate a large number of S-system parameters if they are to be used to infer large-scale network systems containing many network components. Thus, the regression task becomes difficult and time consuming as there is a large parameter space to be optimized with this nonlinear formalism. This is why inference algorithms based on the S-system model have only been applied to small-scale networks. So, to overcome the problems regarding nonlinear models, in this research we have considered a linear model. These models are very simple and can be applied to very large-scale networks. In case of all linear models, the regulatory interactions among the genes are represented by a weight matrix, **W**, where each row of **W **represents all the regulatory inputs for a specific gene. There are mainly two different types of models that lie under this category. These models differ by means of their ability to handle nonlinearity.

There are mainly two entities that are involved in the reverse engineering procedure; a mathematical model of gene regulations and a search method that can find, within the framework of the model, the regulations that are most probable given the dataset of expression levels. In this paper, we have proposed a novel reverse engineering approach for discovering interactions between genes based on time series expression data. This approach builds on the use of a *linear time-variant *formalism to model the gene regulatory network inference problem. Amongst several linear formalisms, the linear time-variant model is the only one which is capable of discovering the nonlinear relationships among genes just like the nonlinear models while dealing with noisy gene expression data. But as compared to nonlinear models, it is simpler and more flexible. This provided a great inspiration towards the present research work to establish a new and faster reverse engineering approach based on the linear model (using computational analysis of microarray dataset) for extracting the relationships among genes clearer. That is, constructing a gene network which resembles a true and accurate genes interaction approach in a genome. As both quantity and quality of experimental data improve, we aim at a more biologically plausible, faithful reconstruction of the target network [10]. This requires adequate inference methods that can handle complex, nonlinear gene models. So, as an inference method, in this work, one of the most promising evolutionary algorithms, *Self-Adaptive *DE has been used to learn the model parameters yielding an optimized network.

System identification using linear time-invariant models [11] simply involves finding an optimal set of parameters for a given model, since the model structure is fixed in advance. However, as compare to the linear differential equations, system identification problems using nonlinear model are time consuming, because not only the parameters of the system but also the system structure must be estimated. Of course, no one claims that there is a linear relationship between the components in a real regulatory network. Instead, the working hypothesis of the linear models is that linear equations can at least capture the main features of the network. But, any model of the system derived using purely linear approaches will, however, not be able to accurately represent the true nonlinear behavior of the real gene network. In order to overcome the above problem, in this paper, linear time-variant systems have been adopted as the model for inferring the gene regulatory networks. That means, nonlinearity has been discovered through a linear model. Although linear time-variant models are still in a linear form, they have a much richer range of dynamic responses than linear time-invariant systems.

**Figure 1:**
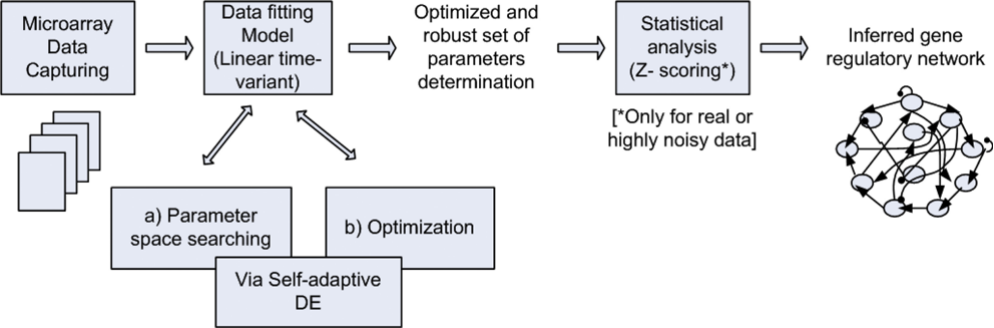
**Workflow of reverse engineering gene regulatory networks**.

Till now there is only one research by Jongrae Kim and Kwang-Hyun Cho who has proposed this model for inferring gene regulatory network [12]. But they used a different method as an optimization engine which includes phase portrait analysis and random perturbations to estimate the model parameters. Their proposal has worked accurately for inferring the real gene regulations but they have not considered the estimation of the model parameters. Their inference model along with the optimization technique involved a large number of computations which were very complex. Also the computational time was great. For large networks, this computational complexity is aggravated. Being inspired by their work, the linear time-variant system has been considered here to model the regulatory network. The current study not only infers the network structure but also estimates this model parameters. To handle complex, nonlinear gene models, Self-Adapative DE has been used to learn the model parameters. To our best knowledge and investigations, this is the first time EA be incorporated with the linear time-variant approach in inferring gene regulatory networks. This is the main contribution of the current study. The overall workflow of this reverse engineering process is given in Figure 1.

The proposed work has been first verified by synthetic data and then its effectiveness is confirmed by analyzing simulated cAMP oscillation data and the real time-series expression data of SOS DNA repair network in E. coli. Specifically, the accuracy of reconstructing gene regulatory networks has been tested with four different cases:

1. Reconstructing gene network from synthetic gene expression data without noise.

2. Reconstructing gene network from synthetic gene expression data with 5% and 10% noise.

3. Reconstructing gene network from the simulated real time-series microarray data (noise-free and 5% noisy) of cAMP oscillations in *Dictyostelium discoideum*.

4. Reconstructing gene network from real time-series microarray data of the well-known SOS DNA repair network in Escherichia coli.

The viability of this work is mainly proved by the synthetic data since the actual structure is unknown for most of the real networks. Nevertheless, the performance is limited to the amount of expression data used and the noise level present in the data. However, for testing the proposed approach, a biomolecular network system has been used which is based on Laub and Loomis model for D. discoideum cAMP oscillation [13]. In that case, almost all the correct and reasonable gene regulations have also been predicted in a very little computational time. Again, the real SOS DNA repair system has also been analyzed and the current work has proved its viability again.

## Results and discussion

In this paper, to confirm the effectiveness of the proposed approach, at first it has been applied to an artificial genetic network inference problem. For this, we have considered both the noise-free and noisy data. Even with the presence of noise, the proposed approach has successfully reverse engineer the network from the synthetic data. Afterwards, this approach is tested using a small size D. discoideum cAMP oscillations model. In this section, the results of these experiments are presented along with sufficient details.

### Inference of a synthetic network with noise free time series data

Here, as the target network we used a small-scale S-system model with the parameter set listed in Table 1[9]. This network was first studied by Tominaga *et al*. in [15] and later many others also tried to infer this network [9,14,16-18]. Hence this network, which represents a typical gene interaction system consisting of 5 genes, has become like a standard network for evaluating the performance of optimization algorithms for the models that can handle nonlinearity. By solving the equation for S-system [8,9], using the rate constants and kinetic orders as given in Table 1, the synthetic time-series data is formed. This synthetic expression data began from initial values which are randomly generated within the range [0.0, 1.0]. As the observed gene expression levels, M = 10 sets of noise-free time-series data are used, each having T = 11 sampling points and covering all five genes of the network. Thus, the observed time-series data for each gene-i consists of 10 × 11 = 110 sampling points.

**Table 1:** S-system parameters for the target network model

*i*	***α***_***i***_	*g*_*i*,1_	*g*_*i*,2_	*g*_*i*,3_	*g*_*i*,4_	*g*_*i*,5_	***β***_***i***_	*h*_*i*,1_	*h*_*i*,2_	*h*_*i*,3_	*h*_*i*,4_	*h*_*i*,5_
1	5.0	0.0	0.0	1.0	0.0	-1.0	10.0	2.0	0.0	0.0	0.0	0.0
2	10.0	2.0	0.0	0.0	0.0	0.0	10.0	0.0	2.0	0.0	0.0	0.0
3	10.0	0.0	-1.0	0.0	0.0	0.0	10.0	0.0	-1.0	2.0	0.0	0.0
4	8.0	0.0	0.0	2.0	0.0	-1.0	10.0	0.0	0.0	0.0	2.0	0.0
5	10.0	0.0	0.0	0.0	2.0	0.0	10.0	0.0	0.0	0.0	0.0	2.0

#### Experimental setup

In accord to the linear time-variant approach, an individual is represented by a candidate set of parameters {*α*, *β*, *ω*, *ϕ*}covering 5 genes. The search regions of the parameters *α *and *β *are set to [0.0, 10.0] and [-3.0, 3.0], respectively. For both *ϕ *and *ω*, the parameter range is set to [-90.0°, 90.0°]. For all the individuals of the population, initially F = 0.5 and CF = 0.9 are considered. Later on, due to the self adaptation, the inference method automatically adjusts these control parameters for each individual. Here, the method has been experimented on the population size 200. The termination criterion for the algorithm is set to the maximum number of generations to run and the maximum generation number is set to 10, 000.

Our algorithm has been implemented in C programming language. The time required for solving a typical run of the associated GRN problem is approximately 4.0 minutes in a PC with 2.4 GHz Intel Pentium IV processor and 512 MB of RAM. The program has been run with the same experimental setup for 10 times (runs).

#### Result

Table 2 shows the parameters {*α*, *β*, *ω*, *ϕ*} estimated by our algorithm on noise-free data sets in a typical run. Using these parameters the weight matrix **W **is obtained by employing Equations 2 and 3. The **W **matrix provides information about relationships among genes and can be used to construct the underlying gene expression network. The inferred W matrix from the noise-free time-series data is shown in the Table 3. As shown in this table, our model has inferred all the true positive regulations. In this case, the sensitivity (*S*_*n*_) and the specificity (*S*_*p*_) averaged over 10 runs are 1 and 0.846, respectively. The MSE between the time-series produced by the underlying model and the observed time-series data as defined by equation 5 is on average 10^-4^. This result demonstrates the strength of the proposed framework in inferring an artificial gene network from noise-free time-series data.

**Table 2:** Sample parameters of the current model obtained using 10 set noise-free data

*i*	*α*_*i*,1_	*α*_*i*,2_	*α*_*i*,3_	*α*_*i*,4_	*α*_*i*,5_	
1	2.545906	2.208793	-2.267179	0.077157	-2.930754	
2	2.999982	-0.645031	2.546457	0.091309	-2.995024	
3	0.695926	-2.137540	0.870462	-1.801902	0.966793	
4	2.992971	2.381830	-2.999674	-0.123073	1.424082	
5	0.558151	1.438226	0.828602	-0.642184	1.274659	

***i***	***β*_*i*,1_**	***β*_*i*,2_**	***β*_*i*,3_**	***β*_*i*,4_**	***β*_*i*,5_**	

1	1.207084	-0.922711	-0.017229	2.991777	-0.534957	
2	-2.999217	2.200550	0.845759	2.043800	-2.798684	
3	2.999671	0.510589	2.997147	-1.854454	2.997597	
4	2.306348	-2.667614	-0.260946	2.921703	-0.854851	
5	-1.743528	-0.658371	2.330255	0.072361	0.196040	

***i***	***ϕ*_*i*,1_**	***ϕ*_*i*,2_**	***ϕ*_*i*,3_**	***ϕ*_*i*,4_**	***ϕ*_*i*,5_**	***ω*_*i*_**

1	-1.236375	-0.066410	-0.433812	-0.139365	0.879294	0.174255
2	-0.703738	-1.251633	0.523514	-1.510473	0.864436	0.085782
3	0.800537	1.569361	-0.148454	1.570789	-0.407212	-0.207031
4	-0.990469	1.438330	0.580545	1.328800	1.128653	-0.465122
5	1.257577	-1.570644	-1.203923	0.401359	1.452560	-0.186500

**Table 3:** Weight matrix estimated using 10 sets noise-free time series data

Gene	1	2	3	4	5
1	-1.854097	-0.814807	1.806863	0.0	-1.704923
2	3.507204	-1.940482	0.0	0.0	0.0
3	40.0	-1.220429	-2.928485	0.0	0.0
4	-0.709594	0.0	1.646988	-2.027261	-2.104541
5	0.0	0.0	0.0	3.136715	-2.113481

### Inference of a synthetic network with noisy time series data

The real challenge lies on the capability of the inference algorithm in constructing the network from noisy data. We have also analyzed the performance of our proposed approach by conducting the experiments with the set of 5% and 10% noisy time series data. In all the cases discussed, the current work requires 4.16 minute to predict the GRN which is very small time on the above-mentioned PC configuration.

#### Experimental setup

As the target model we select the same network used in previous experiment with the same target parameter set. Data points were generated using the same sets of initial expression used in previous experiment. Two different experiments have been conducted along with 10 different 5% and 10% noisy data sets. Like before, 11 sampling points from each of these 10 time-series data sets have been used for optimization. Both of these experiments are also conducted with 10 runs using the similar setup described in the previous section.

#### Results for 5% noisy time series data

In a typical run, the weight matrix **W **inferred on this experiment is presented in Table 4. Even using these 5% noisy data, the proposed approach has proved its success in identifying all the correct gene regulations. Only three false positive regulations namely gene-4 → gene-2, gene-5 ⊣ gene-3 and gene-2 ⊣ gene-5 have been identified. No false negative regulation has been inferred in this aspect. Here, *S*_*n *_and *S*_*p *_are 1 and 0.769, respectively. The MSE is approximately 0.56.

**Table 4:** Weight matrix estimated using 10 sets 5% noisy time series data

Gene	1	2	3	4	5
1	-2.409106	0.0	1.480375	0.0	-2.819197
2	2.865662	-2.509981	0.0	1.169968	0.0
3	50.0	-0.922008	-3.095746	0.0	-0.758363
4	0.0	0.0	1.109536	-3.375679	-1.609716
5	0.0	-0.669722	0.0	3.755632	-1.840754

#### Results for 10% noisy time series data

**Figure 2:**
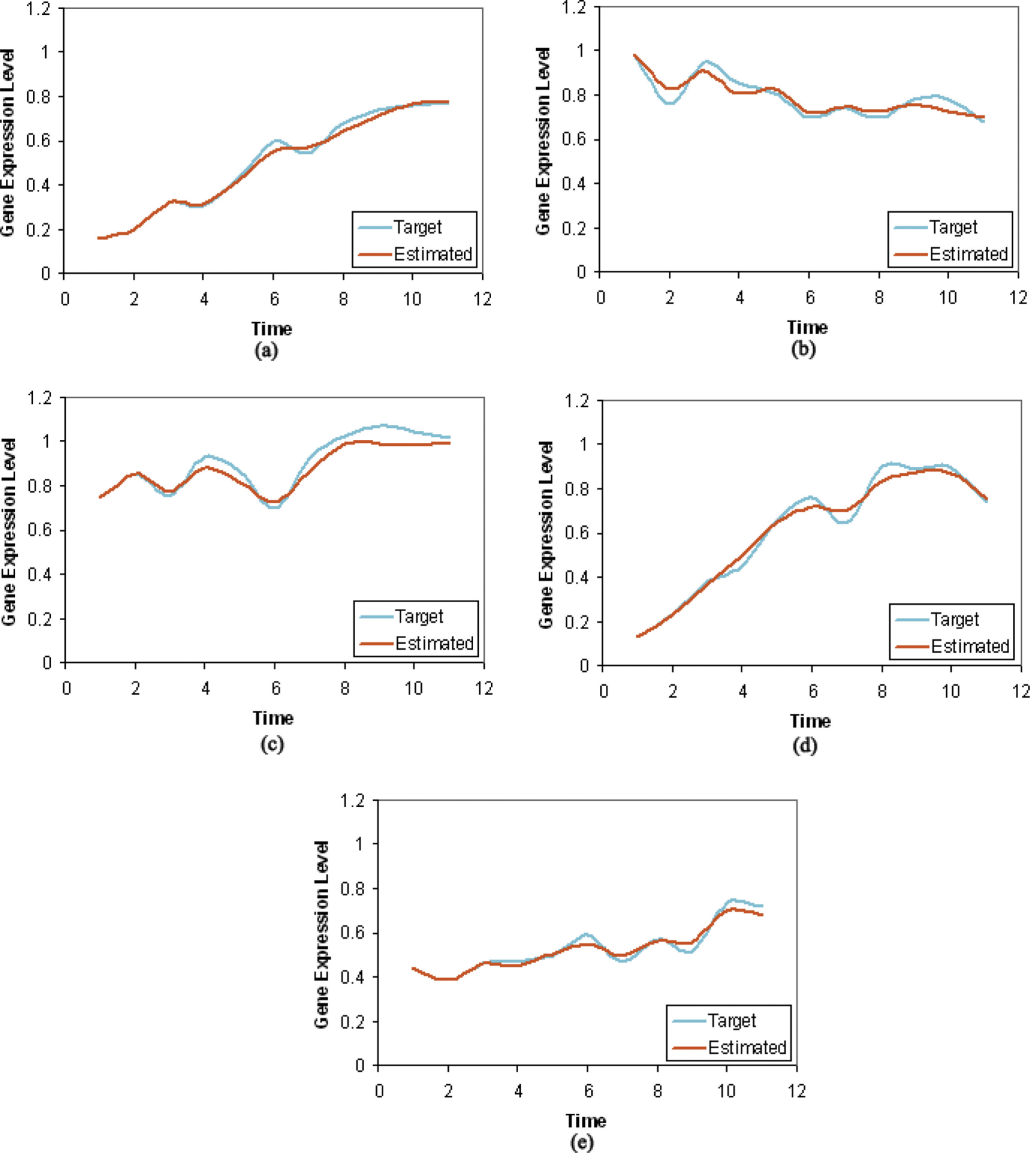
**The target time-series and the estimated time-series data of 10% synthetic noisy data**.

The weight matrix **W **inferred in a typical run using these 10% noisy time-series data is presented in Table 5. In this case, the present work have proved its strength by recognizing almost all the true positive gene regulations. Here, four false positive regulations namely gene-4 ⊣ gene-1, gene-4 → gene-2, gene-5 ⊣ gene-3 and gene-1 → gene-5 have been identified. But, our approach has missed the true regulation gene-1 → gene-2. Thus, the estimated *S*_*n *_and *S*_*p *_averaged over all 10 runs are 0.916 and 0.667, respectively. The MSE is approximately 2.75 by considering the average of all such values on 10 runs. The observed and estimated time dynamics of all 5 genes obtained in this case is shown in Figure 2.

**Table 5:** Weight matrix estimated using 10 sets 10% noisy time series data

Gene	1	2	3	4	5
1	-2.833573	0.0	1.376733	-0.584010	-2.106859
2	0.0	-2.150746	0.0	0.894012	0.0
3	60.0	-1.143628	-2.878722	0.0	-0.755654
4	0.0	0.0	1.419933	-3.079639	-1.811521
5	0.776600	0.0	0.0	2.844820	-2.636815

### In silico example: Dictyostelium discoideum cAMP oscillations

Dictyostelium discoideum cells signal each other by emitting stable oscillations of cAMP at the beginning of the aggregation phase of their development. The oscillations continue during chemotaxis towards higher gradients of cAMP concentration as D. discoideum aggregate to survive. In 1998 a model for the biomolecular network underlying the stable oscillations in cAMP was postulated by Laub and Loomis [13]. The corresponding non-linear differential equations are given in [13]. In this case, to control the overall oscillation mechanism, 7 different genes are involved, namely ACA, PKA, ERK2, RegA, cAMPi, cAMPe and CAR1. Pulses of cAMP are produced when ACA is activated after the extracellular cAMP (cAMPe) binds to the surface receptor CAR1. When internal cAMP (cAMPi) accumulates, it activates the protein kinase PKA. Ligand-bound CAR1 also activates the MAP kinase ERK2. When RegA hydrolyses the internal cAMP, PKA activity is inhibited by its regulatory subunit and the activities of both ACA and ERK2 go up. The connections among many of the above components are shown in [13]. Here, only those activities that are regulated, either directly or indirectly, by other activities in the circuit are included.

#### Experimental setup

The expression data of this cAMP oscillation have been generated using Loomis's model [13]. These data have been normalized within the range (0, 1]. In this case, the search regions of the parameters *α *and *β *are set to [-5.0, 5.0] and [-3.0, 3.0], respectively. For both *ϕ *and *ω*, the parameter range is set to [-90.0°, 90.0°]. The above 7 genes are monitored with 10 instants in 5 data sets. That is, having 10 measurements in each data set, there exists 10 × 5 = 50 sampling points for each gene. In total 10 runs have been carried out to assure the statistical significance of the search method. All of the other experimental conditions are the same as those used in the experiments discussed in the previous section.

#### Results

Here, we have simulated noise-free and 5% noisy cAMP oscillation expression data. In different runs of these experiments, different regulations are being predicted. So Z-score (average/standard-deviation), a statistical approach has been applied to analyze which regulations are more significant and less diverse than others [1]. Only those regulations have been considered whose Z-score value be above the threshold Z_*th *_= 1.0. The value of this threshold was set empirically.

**Figure 3:**
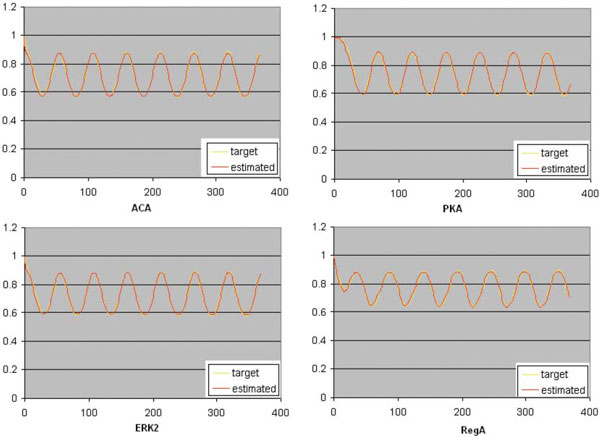
**Target versus estimated time-series data computed from the obtained model on the experiment of the cAMP oscillation with noise-free data**.

In a typical run, the inferred weight matrix **W **from the noise-free oscillation data, is presented in Table 6. The present work has proved its strength by recognizing all the true positive regulations along with 3 false positives. The estimated *S*_*n *_and *S*_*p *_averaged over all 10 runs are 1.0 and 0.811, respectively. The MSE is approximately 0.051622 by considering the average of all such values on 10 runs. The observed and estimated oscillation of all 7 genes obtained by the underlying method is shown in Figure 3.

**Table 6:** Weight matrix estimated using 5 sets noise-free time series data for cAMP oscillation

Gene	ACA	PKA	ERK2	RegA	cAMPi	cAMPe	CAR1
ACA	-2.717321	-2.214067	0.0	0.0	0.0	0.0	1.232814
PKA	0.0	-2.432351	0.0	0.0	1.932617	-1.845066	0.0
ERK2	0.0	-1.712449	-2.007182	0.0	0.0	0.0	1.310419
RegA	0.0	0.0	-1.101286	-3.216777	0.0	0.0	0.0
cAMPi	1.972690	7.0	-1.120288	-1.930469	-1.212059	0.609732	0.0
cAMPe	1.700202	0.0	0.0	0.0	0.0	-1.833212	0.0
CAR1	0.0	0.0	0.395086	-0.0	0.0	2.227322	-1.568279

Lastly, the inferred weight matrix **W **from the 5% noisy oscillation data is presented in Table 7. The current work has proved its potency by inferring almost all the true positive regulations in this regards along with 4 false positives. In this case, *S*_*n *_and *S*_*p *_are 1.0 and 0.771, respectively. The MSE is approximately 1.31961 averaged over 10 runs.

**Table 7:** Weight matrix estimated using 5 sets 5% noisy time series data for cAMP oscillation

Gene	ACA	PKA	ERK2	RegA	cAMPi	cAMPe	CAR1
ACA	-1.778255	-2.945847	0.0	0.0	0.0	1.132729	0.845161
PKA	0.0	-1.827042	0.0	0.0	2.435239	-1.857534	0.0
ERK2	0.825868	-3.404052	-1.851645	0.0	0.0	0.0	1.668507
RegA	0.0	0.0	-2.424868	-2.846192	0.0	0.0	0.0
cAMPi	1.161844	0.0	0.0	-1.209490	-1.585139	1.648658	0.0
cAMPe	2.631136	0.0	0.0	0.0	0.0	-1.683819	1.190072
CAR1	0.0	0.0	0.0	-0.0	0.0	2.795634	-2.961315

Along with correct predication, the main feature of the proposed approach is its less computational time. It requires 2.46 minute to infer the GRN of cAMP oscillation on the above-mentioned PC configuration. This is so little as compared to many other works.

### Analysis of real microarray data

**Figure 4:**
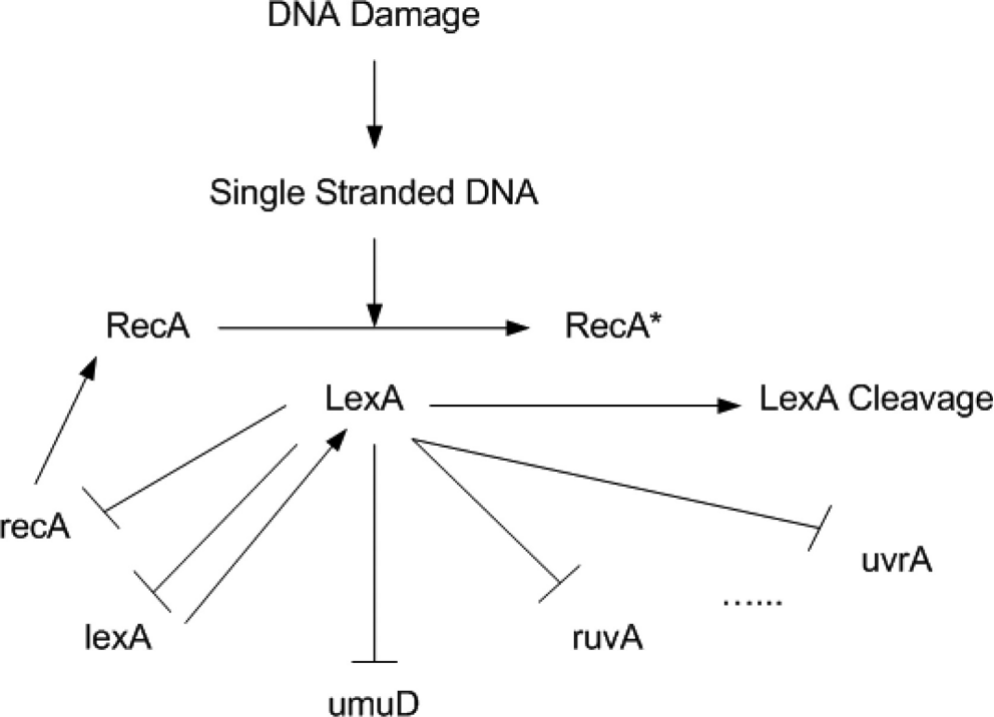
**The bacterial E. coli SOS DNA Repair network. Activations are represented by →, while supression by ⊣. Genes are in lower cases, proteins in capital letters**.

The success on the experimentations of synthetic network and cAMP oscillation leads to the experiment to see how well the approach works in reconstructing network topology and estimating system parameters from real microarray data. In this regards, the proposed method has been applied to analyze the well-known SOS DNA repair network in *Escherichia coli *as shown in Figure 4. This gene regulatory network is well known for the responsibility of repairing the DNA after some damage. It is the largest, most complex, and best understood DNA damage-inducible network to be characterized to date. The entire system involves more than 100 genes [19]. But only 30 genes serve as key regulators at the transcriptional level. The expression of the genes in the SOS regulatory network is controlled by a complex circuitry involving the RecA and LexA proteins. In a normal state, the LexA acts as the master repressor of more than 20 genes, including *lexA *and *recA *genes. This repressing is done by its binding to the interaction sites in the promoter regions of these genes. When DNA damage occurs, one of the SOS proteins, RecA, acts as a sensor. By binding to single-stranded DNA, it becomes activated, senses the damage and mediates LexA autocleavage. The drop in LexA levels, in turn, halts the repression of the SOS genes and activates them. When the damage has been repaired, the level of activated RecA drops and it stops mediating LexA autocleavage. LexA in turn accumulates and represses the SOS genes, and cell returns to its initial state [14].

#### Experimental setup

The expression data sets of the SOS DNA repair system have been downloaded from the homepage of Uri Alon Lab [20]. These data are expression kinetics of 8 genes namely *uvrD*, *lexA*, *umuD*, *recA*, *uvrA*, *uvrY*, *ruvA *and *polB*. Four experiments are done for various UV light intensities (Exp. 1 and 2:5 *Jm*^-2^, Exp. 3 and 4:20 *Jm*^-2^). In each experiment, the above 8 genes are monitored with 50 instants evenly spaced by 6 minutes intervals.

In this research, only 6 genes have been chosen from Alon's experiment data, i.e., *uvrD*, *lexA*, *umuD*, *recA*, *uvrA *and *polB*. This subnetwork is a well studied one and interactions among different genes are known. The same set of genes were used by Cho [21] and kimura [17,22] and their method successfully inferred the regulatory network of the selected genes. Their success has motivated us to select the same genes in the present study. This gives a chance to compare different approaches and see how the proposed method works. Although Alon's experimental data contains 4 sets of time-series expression levels, here we have considered data from experiment 3 and 4. That is, having 50 measurements in each data set, there exists 50 × 2 = 100 sampling points for each gene including the initial concentrations which are all zeros. Here, these initial concentrations have been removed from both of the data sets, since the underlying model cannot produce different time courses from same initial conditions. That is, each of the considered 6 genes has 49 × 2 = 98 sampling points. The data corresponding to each gene has been normalized within the range (0, 1] against their maximum value. All the zero expression levels in these two data sets have also been replaced with a very small value (around 10^-4^). The search regions of the parameters *α *and *β *are set to [-20.0, 20.0] and [-5.0, 5.0], respectively, whereas *ω *and *ϕ *are set to the same value as used in the previous experiments. In total 10 runs have been carried out to assure the statistical significance of the search method. All of the other experimental conditions are the same as those used in the experiments discussed in the previous section.

#### Results

**Figure 5:**
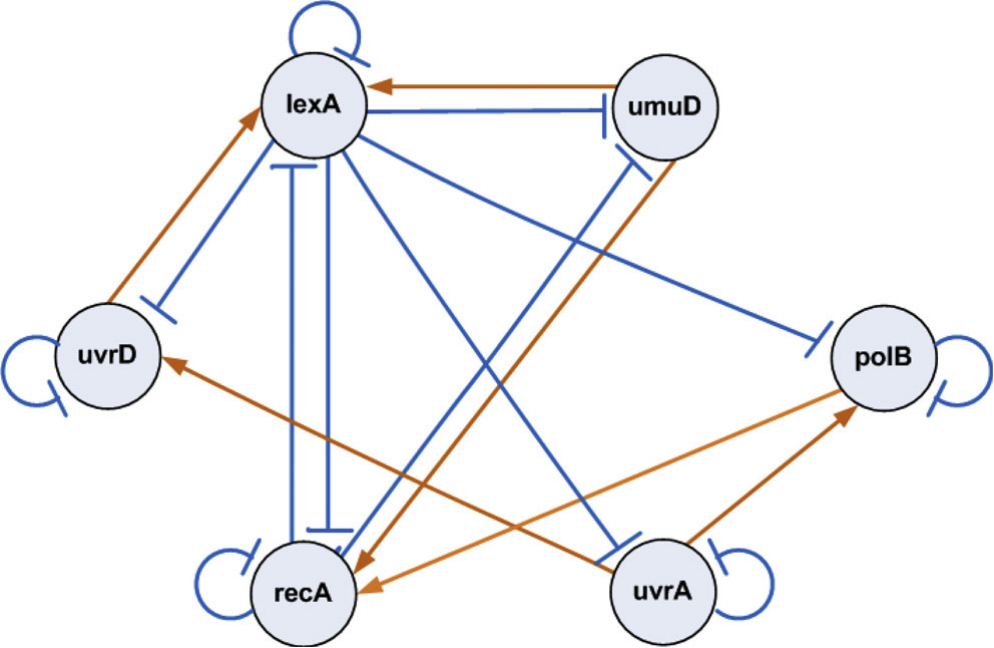
**The 6-gene SOS repair network structure inferred by the proposed approach**. [⊣ = *supression *and → = *activation*]

As the input data is from actual microarray experiment, there is noise present in that. No body knows how much noise is inherent in these data. These data may have had an influence on the inference algorithm. In this research, due to this noise level, the results have been much dispersed. In different runs of the experiment, different regulations have been predicted. Only those regulations have been considered whose Z-score value be above the threshold Z _*th *_= 1.0. The corresponding SOS network structure inferred by the proposed approach is shown in Figure 5.

**Figure 6:**
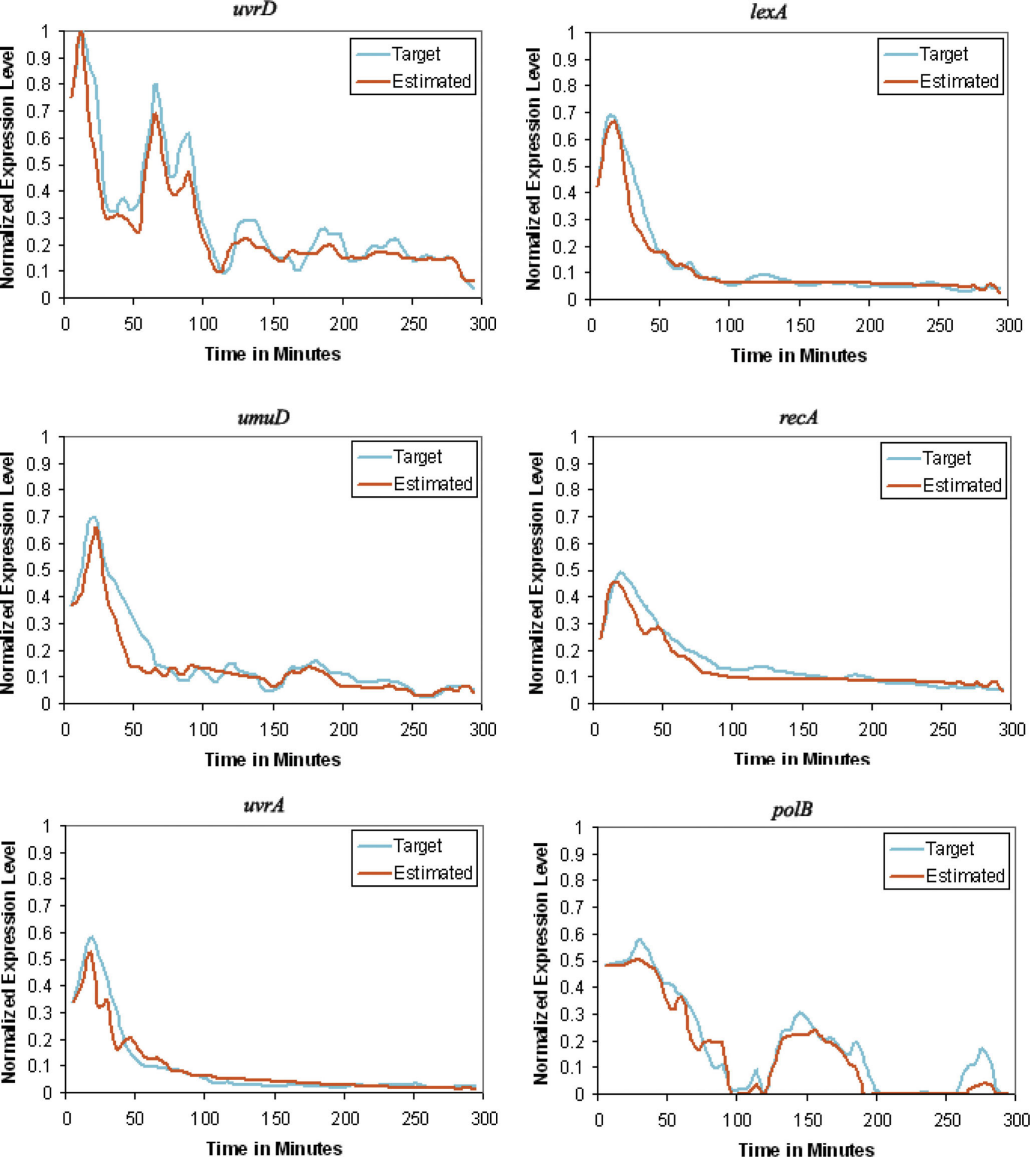
**Target versus estimated time-series data computed from the obtained model on the experiment of the SOS DNA repair system**.

The observed and estimated time dynamics of all 6 genes in the SOS system obtained by the underlying method is shown in Figure 6. It confirms that the proposed approach recovers most of the true dynamics of the original system. The inferred network for the SOS system has contained some reasonable regulations. According to Figure 5, the negative regulations from *lexA *to *uvrD*, *lexA*, *umuD*, *recA *and *uvrA *have been successfully identified. The regulation of *lexA *from *recA *have also been correctly identified. Regulation of *lexA *by *umuD *is also known. The regulation of *umuD *from *recA*, inferred by the proposed method, also appears to be reasonable, as it is contained in a network now known [19]. These inferred true regulations have proven the strength of the proposed work in inferring real gene network topology. The reconstruction algorithm misses the known regulation *recA *⊣ *uvrA *[14]. Reason behind these possible failures may be due to inadequate number of data sets and noise present in data. Some other regulations have been inferred, those are either novel regulatory pathways or false-positive findings.

Table 8 shows the summary of the known regulations and predicted regulations for all the 6 genes in the SOS repair network identified by the proposed algorithm. The number of correct regulations inferred by the proposed work is larger than that of the S-tree based method proposed by Cho [21], S-system based method proposed by Perrine [14] and NGnet model proposed by Kimura [17]. Although some of the inferred regulations have not been experimentally observed, some might be new findings and the rest should be false-positive. In order to infer a more reliable network, more sets of expression data obtained from additional biological experiments or prior knowledge about the target system need to use. The computational time of the proposed work for inferring this SOS network is much shorter. It has obtained SOS network in approximately 4.36 minutes on a single-CPU personal computer (Pentium IV 2.4 GHz). Whereas the S-tree based system [21] running on the computer system Athlon Mp2800+ took about 35 hour with 2 × 10^6 ^generations to infer this network. The method proposed in [17] took 47.1 sec × 6 = 4.7 minute on a single-CPU personal computer (Pentium IV 2.8 GHz) in this aspect.

**Table 8:** Predicted gene regulations for SOS DNA repair system

Gene	Predicted Regulations	References
*uvrD*	uvrD ⊣ uvrD, lexA ⊣ uvrD, uvrA → uvrD	[21,17,22]
*lexA*	uvrD → lexA, lexA ⊣ lexA, umuD → lexA, recA ⊣ lexA	[31,21,17]
*umuD*	lexA ⊣ umuD, recA ⊣ umuD	[31,19,22]
*recA*	lexA ⊣ recA, umuD → recA, recA ⊣ recA, polB → recA	[31,17]
*uvrA*	lexA → uvrA, uvrA ⊣ uvrA	[22]
*polB*	lexA ⊣ polB, uvrA → polB, polB ⊣ polB	[22]

Since it is difficult for the proposed method to control the number of regulations inferred, the obtained networks would generally contain a number of false positive regulations. To influence biologists to use the results obtained from this method, it is needed to ensure the reliability of the inferred regulations which are unknown. In a future work, the proposed method will be modified for this purpose.

## Conclusion

The performance of the proposed framework makes it more applicable to the problem of reverse engineering of gene networks. Amongst reverse engineering approaches linear time-variant model is of particular interest as it is capable of discovering the nonlinear relationships among genes like other nonlinear formalisms while dealing with noisy gene expression data. To infer an optimized network structure, here DE is used as the optimization engine. The proposed framework has been first verified by synthetic data and then its effectiveness is confirmed by analyzing simulated cAMP oscillation data and the real time series expression data of SOS DNA repair system. In real network analysis, the present work has been succeeded in finding several reasonable regulations as compare to the other existing methods. In all the cases, even with the presence of noise, the current work has inferred almost all the correct regulations. Thus, along with some future enhancements this work can boost systems biology research.

## Methods

### Modelling nonlinearity using linear time-variant approach

For a gene regulatory network consisting of n genes, the mathematical formalism of this model is given by the following equation:(2)

Here, *Z*_*i*_(*t*) be the total regulatory input to gene-i, *X*_*i *_is the level of expression of gene-i at time t. The **W **matrix provides information about relationships among genes and can be used to construct the underlying gene expression network. The weight coefficient *W*_*i*, *j *_indicates the strength of the influence of gene-j on the regulation of gene-i and is the respective element of the transition matrix **W**. A positive value of *W*_*i*, *j *_means gene-j is inducing gene-i whereas a negative value is an indication of repression. On the other hand, a zero value on **W **indicates that gene-j does not influence the transcription of gene-i. Equation 2 shows that *W*_*i*, *j *_is a time-varying function. Here, *W*_*i*, *j*_(*t*) can be written as a finite sum of Fourier series [24] given as the following.(3)

Here, *α*_*i*, *j*_, *ω*, *ϕ*_*i*, *j *_and *β*_*i*, *j *_are the constants to be determined for *i *= 1, 2, ......, *n *and *j *= 1, 2 ......, *n*. These values are the model parameters. Thus the linear time-variant model is defined by the parameters set ={*α*, *β*, *ω*, *ϕ*}. In equation 3, *β*_*i*, *j *_represents the linear part of the interactions and the sinusoidal term approximates the nonlinear terms in the interactions.

The response of the gene-i to the regulatory input is the expression level at time *t *+ 1, i.e. *X*_*i*_(*t *+ 1). Thus, the value of *X*_*i*_(*t *+ 1) is obtained by normalizing *Z*_*i *_by using the following "squashing" function:(4)

where the value of *X*_*i*_(*t *+ 1) lies between 0 and 1.

### Model evaluation criteria

In the current study, the gene network estimation problem has been formulated as a function optimization problem based on a linear time-variant approach. In an optimization problem, one is more interested to find the network that best fits the experimental data in the exploration of the search space. However, when adequate time-series expression values of relevant genes are given, a set of parameter values *α*, *β*, *ω *and *ϕ*, in many cases, will not be uniquely determined. The reason behind this is that it is highly possible for the other sets of parameter values showing the similar time-course. Therefore, even if one set of parameter values that matches the observed time-series is obtained, this set may be only one of the best candidates that explains the observed time-series values. The main strategy is to explore and exploit these candidates within the huge searching space of parameter values. For searching an optimal set of parameters for gene networks, the most commonly used fitness evaluation criterion is to compute the difference between the calculated expression levels and the observed experimental dynamics. This is termed as *Mean Squared Error*(MSE) which was introduced by Tominaga *et al*. [15] and later used by others [9,16-18]. The smaller the value of the MSE function, the better the match between observed and calculated expression dynamics. A single set of time series data is not sufficient to identify a unique solution. So, to capture the overall behavior of the complex and nonlinear GRNs, it is better to use multiple sets of time series data [25]. Thus, for each set of parameters representing regulation networks in linear time-variant system, the MSE based fitness evaluation function using multiple sets of dynamics becomes:(5)

where *X*_*k*, *i*, *exp*_(*t*) and *X*_*k*, *i*, *cal*_(*t*) represent the experimentally observed and numerically computed expression level of gene-i in the k-th data set at time t, respectively.

### Inference method

Because of nonlinearity in gene regulations and curse of dimensionality, genetic algorithm can be applied to approximate the optimal solution in the solution space and can learn the network structure. Here, as the GA, the Self-adaptive version of Differential Evolutionary (DE) method [26] has been used. Experimental results have shown that, DE as well as it self-adaptive version converges faster and with more certainity than many others acclaimed global optimization methods and also proven its effectiveness in genetic network inference [27]. Because of these promising properties DE has been chosen as the optimization tool in this paper to optimize the fitness function defined in equation 5.

Differential Evolution (DE) is a simple population-based, stochastic search algorithm for global optimization created by Ken Price and Rainer Storn [28]. From 1994, DE has been used for many optimization problems and has proved its robustness and effectiveness [18,29,30]. This is an iterative algorithm, where the successive generations try to get an optimal solution, stopping when the maximum number of generations is reached or when the fitness of the current solution is greater than a predetermined value. It utilizes NP D-dimensional parameter vectors:(6)

as a population for each generation. Here, NP is the total number of individuals in a population and G denotes the generation. NP does not change during the optimization process. The initial vector population is generally chosen randomly between the lower (**X**_*i*, *low*_) and upper (**X**_*i*, *upp*_) bounds defined for each variable **X**_*i *_to cover the entire parameter space. This bounds are specified by the user according to the nature of the problem.

Following the initialization phase, DE performs several vector operations in a process called *evolution*. In this evolution process, there are mainly three operations that are associated with DE at each generation to produce the next generation: *mutation*, *crossover *and *selection *[28]. During each generation of operation, DE employs both mutation and crossover to produce a *trial vector*, **U**_*i*, *G *_for each *target vector*, **X**_*i*, *G*_. Then a selection operation takes place in order to pick the better of trial and target vector based on their fitness and after that the better vector is placed as a next generation individual. Each population vector has to serve once as the target vector so that NP competitions take place in one generation. The formalisms of these three operations of DE are described in [28].

Although DE has been shown to be a powerful evolutionary algorithm for global optimization, users are still faced problem related to the hand-tuning of the evolutionary control parameters which are the main factors of the optimization problem. As a solution, Janez Brest has proposed jDE, a self-adaptive version of DE, for enhanced convergence property and robustness [26]. It is same as DE; i. e. it also utilizes the mutation, crossover and selection processes accept with one modification. It uses self-adaptive mechanism on control parameters F and CR. In this case, along with the D dimensional parameters of the problem, a crossover factor CR and an amplification constant F are also included as parameters at individual levels. The values of these parameters are adaptively calculated. The strategy behind this self-adaptation is that, the better values of these control parameters lead to better individuals, which, in turn, are more likely to survive and produce offspring; thereby, can propagate these better parameter values.

According to the self-adaptation rules, at each generation, the new control parameters *F*_*i*, *G*+1 _and *CR*_*i*, *G*+1 _for *ith *individual can be calculated as follows:(7)(8)

Randomly, the new F takes a value within the range [0.1, 1.0] whereas the new CR takes the value within the range [0, 1]. Here, *rand*_*j*_, for *j *∈ {1, 2, 3, 4} are randomly generated values within the range [0, 1]. Both *τ*_1 _and *τ*_2 _have value 0.1 which represent probabilities to adjust control parameters F and CR. Again, *F*_*low *_and *F*_*upp *_have values 0.1 and 0.9 respectively. *F*_*i*, *G*+1 _and *CR*_*i*, *G*+1 _are obtained before the mutation operation of DE is performed [27]. So they can influence the mutation, crossover and selection operations of each individual at each generation and can produce a better individual **X**_*i*, *G*+1 _for the next generation population.

### Algorithm

The inference strategy of the GRN problem is an iterative process. Here, each iteration is called a generation. The entire set of generations is called a run. At the end of a run, we expect to find a more highly fit individual with optimized parameter set which can be used to represent the correct network structure. In summary, the overall inference procedure i.e. the reverse engineering algorithm of this research work for estimating the parameters of the linear time-variant model (representing the GRN problem) is given as follows:

### Inference-Algorithm()

1. Initialize the population *P*_*G *_(NP individuals) covering the whole search space with candidate solutions, where G = 1. Here, each individual with parameters {*α*, *β*, *ω*, *ϕ*} is randomly generated.

2. Evaluate the fitness value of each individual using equation 5.

3. Generate the next generation population *P*_*G *_+ 1 with candidate solutions using self-adaptive DE.

(a) Choose target individual.

(b) Calculate the new CR and F for each individual using equations 7 and 8.

(c) Randomly choose 3 different population members. For each individual **X**_*i*, *G*_, *i *= 1, 2 ....., *NP*, employing these selected members, a new individual **V**_*i*, *G*+1 _is generated using the mutation operation of DE described in [28].

(d) Do crossover of *V*_*i*, *G*+1 _with target individual *X*_*i*, *G *_to get trial individual *U*_*i*, *G *_using equations as described in [28]. If any parameter of this trial individual goes off the boundary of parameter range, then it is set to boundary value using the equations in [26].

(e) Compare the fitness values of the trial and the target individual and keep the better one in the next generation.

(f) Go to step 3(a) and continue the whole process until the size of the next generation population is equal to NP.

(g) Replace the current population by the next generation population.

4. If there have been 10, 000 evaluations, then stop. Otherwise set *G *= *G *+ 1 and go to Step 3.

Note that, this algorithm will run until the generation is 10, 000. The number of generations can be more than 10, 000, but according to our investigation throughout this research work, then the convergence rate is almost same as that of employing 10, 000 generations.

## Competing interests

The authors declare that they have no competing interests.

## Authors' contributions

MK has implemented the proposed algorithm and performed the experiments. MK and NN have proposed the approach. NN and HI supervised the whole work. All authors have read and approved the manuscript.
